# Is a Prolonged Emergency Department Stay a Risk for Patient Safety?

**DOI:** 10.1016/j.acepjo.2025.100170

**Published:** 2025-05-28

**Authors:** Anna Eidstø, Jalmari Tuominen, Jari Ylä-Mattila, Heini Huhtala, Ari Palomäki, Teemu Koivistoinen

**Affiliations:** 1 Department of Emergency Medicine, Tampere University Hospital, Tampere, Finland; 2 Faculty of Medicine and Health Technology, Tampere University, Tampere, Finland; 3 Department of Emergency Medicine, Kanta-Häme Central Hospital, Hämeenlinna, Finland; 4 Faculty of Social Sciences, Tampere University, Tampere, Finland

**Keywords:** emergency department, emergency department length of stay, EDLOS, mortality, crowding

## Abstract

**Objectives:**

The emergency department (ED) length of stay (EDLOS) has been studied as a metric for ED crowding, as well as an individual risk factor for patient outcomes. Although the results from prior studies vary, larger studies seem to show an association between a prolonged EDLOS and increased adverse events. We aimed to determine how a prolonged EDLOS impacts short-term mortality and to concurrently study these outcomes in different ED occupancy situations.

**Methods:**

A retrospective, single-center cohort study was conducted in the Tampere University Hospital ED, Finland. The study sample included 58,440 ED visits leading to hospital admission between January 2018 and February 2020. The main outcome measure was 10-day all-cause mortality in relation to each hour spent in the ED. Various potential confounding factors were taken into consideration, including patient-specific and ED performance-related variables. Patients were divided into 2 subgroups based on whether they were exposed to crowding during the ED stay or not, with crowding defined as the ED occupancy ratio exceeding 90%.

**Results:**

The 10-day mortality rate was 1.6% (n = 938). A prolonged EDLOS was not associated with mortality in adjusted logistic regression analyses (odds ratio for 1-hour increase in EDLOS, 1.01; 95% CI, 0.99-1.04). Examining the crowded and noncrowded subgroups did not change the findings.

**Conclusion:**

In a Finnish tertiary hospital, a prolonged EDLOS was not an individual risk factor for increased 10-day mortality despite adjusting for confounding factors and considering the possible crowding status of the ED.

**The Bottom Line**

The aim of this study was to clarify the effect of emergency department length of stay on patients’ short-term mortality. The subject is highly complicated and easily confounded by crowding, as well as patient-specific factors, including the severity of the illness and prior comorbidities. This study was conducted in a Finnish tertiary hospital and included 58,440 visits in a 2-year study period. We sought to adjust the analyses for relevant confounding factors; as a result, a prolonged stay did not seem to stand out as an individual risk factor for 10-day mortality among admitted patients.

## 1 Introduction

### 1.1 Background

Exposure to emergency department (ED) crowding has been associated with a lower quality of care and adverse patient outcomes.^1^, ^2^, ^3^, ^4^, ^5^, ^6^, ^7^ High occupancy has also been associated with a longer ED length of stay (EDLOS), even among high-acuity patients.^8^ EDLOS has been used as a metric to demonstrate ED crowding,^9^ and it has also been studied as an independent risk factor for patient outcomes.^10^, ^11^, ^12^, ^13^ However, the association between a prolonged EDLOS and adverse outcomes remains controversial.

A major study from the UK showed a somewhat linear correlation between EDLOS and standardized mortality ratio after 5 hours, even after adjusting for ED crowding status and patient comorbidities.^10^ In contrast, neither Balen et al^11^ nor Asheim et al^12^ found an association between EDLOS and mortality in their studies, which were also adjusted for factors indicating ED crowding. A review study published in 2022 was unable to conclude whether a prolonged stay posed a safety hazard for patients in general, but recommended paying special attention to the elderly.^14^ Closely related to EDLOS, there is some evidence that longer boarding times (ie, patients waiting to be admitted to an in-hospital bed) might be associated with adverse patient outcomes.^15^^,^^16^

### 1.2 Importance

It has been comprehensively described in the literature that ED crowding is associated with short-term mortality,^2^, ^3^, ^4^, ^5^^,^^17^^,^^18^ as also demonstrated in our recent study.^19^ These results strongly suggest that EDs should pay attention to avoid the highest peak occupancies; yet, it remains unclear whether a prolonged EDLOS is associated with excess mortality.

### 1.3 Goals of This Investigation

The aim of this study was to investigate (1) the impact of an EDLOS on 10-day mortality and (2) whether these results vary at different levels of ED occupancy.

## 2 Methods

### 2.1 Study Design and Setting

A retrospective single-center observational study was conducted in the ED of Tampere University Hospital, Finland. One of the largest EDs in the Nordic European Countries, it provides secondary care for more than 500,000 residents in the Pirkanmaa Hospital District and is the only hospital in the region that manages all severe emergency situations. In addition, the hospital is a tertiary care unit for a catchment area of over 900,000 residents. The treatment of patients under 16 years of age without an acute traumatic injury was gradually taken over by the hospital’s Pediatric Unit from September 2018 to 2019. Thus, the study population mostly consisted of adults (only 671 children under 16 years were included).

The study was approved by the hospital’s Research Director (R22601). The Strengthening the Reporting of Observational Studies in Epidemiology guidelines were also applied to this study.^20^

### 2.2 Participants and Measurement

We included ED visits from January 2018 to February 2020. The following variables were collected for each patient visit: personal identity code, sex (male/female), age at arrival (years), date and time of arrival and discharge, necessary International Classification of Diseases 10th Revision (ICD-10) coded diagnoses from 10 years prior to the visit,^21^ emergency medical services (EMS) transport, Emergency Severity Index (ESI) triage classification,^22^ admission or nonadmission to a hospital, whether the patient died within 10 days after the ED visit, and the date of death. Patients’ specialties were categorized as conservative (medicine, neurology, psychiatry, and oncology), operative (orthopedic, surgery, ear-nose-throat, and gynecology), or other (ophthalmology and undetermined), depending on the main complaint at arrival. The included study cohort consisted of bed-occupying patients who were admitted to a hospital for follow-up care but were not primarily treated in the resuscitation room. Patients who were confirmed dead upon arrival or redirected by triage were excluded, as well as a few patients who never arrived at the ED (eg, phone calls). If there was >1 visit within 10 days prior to a patient’s death, only the most recent visit was chosen for observation, and the prior visits were considered as visits without following death. The flowchart contains more detailed information (Fig).

**Figure:**
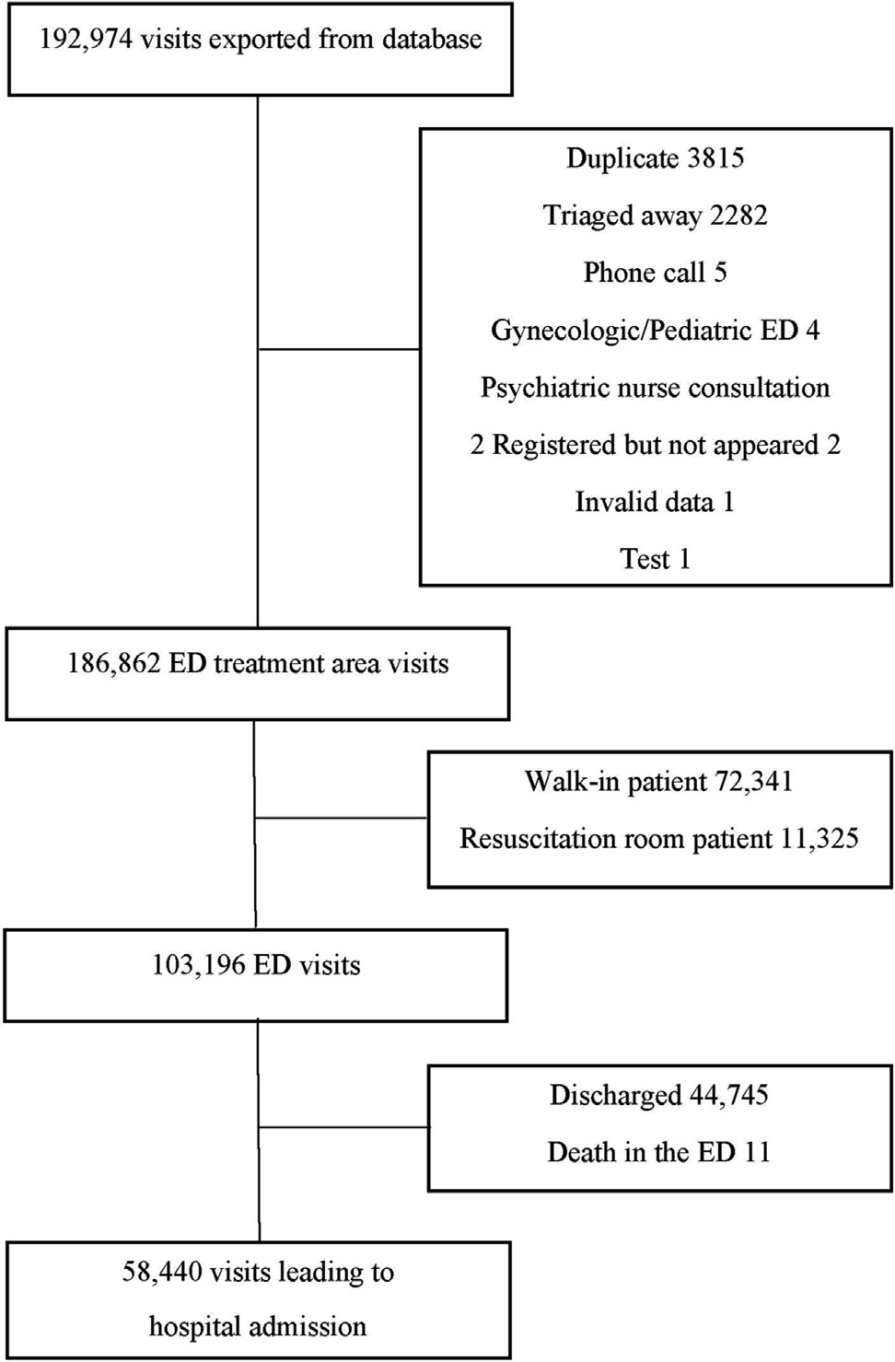
The study selection flowchart. ED, emergency department.

The shift for each visit was defined as the time of arrival (day 8 am to 3.59 pm, evening 4 pm to 10.59 pm, and night 11 pm to 7.59 am). Although we were unable to collect the actual staffing resource from the database, the staffing levels mostly vary between the times of day and are covered in the shifts. The EDLOS was measured from the patient’s arrival at the ED until their discharge to a hospital ward. It was not possible to reliably differentiate between ED waiting times and boarding.

The highest temporal peak occupancy during each visit was calculated based on the registered arrival and discharge times. We used the ED occupancy ratio (EDOR) to accurately measure the level of crowding.^23^ The occupancy ratio is calculated by dividing the patients by available beds; thus, a value of 1.0 means 100% occupancy. This ED has a total of 65 beds and an additional 10 to 12 hallway beds. However, the extra hallway beds are not calculated in the bed count because their usage indicates overflow in the ED, and the EDOR should be >1.0. Furthermore, the study population was divided into 2 groups based on whether the patient was exposed to crowding, defined as maximum EDOR (EDOR_MAX_) ≥ 0.90 during the ED visit. This threshold was selected based on our previous work showing an association between EDOR > 0.90 and 10-day mortality.^19^ All visits experiencing even 1 minute of EDOR_MAX_ ≥ 0.90 were considered “crowded.”

### 2.3 Statistical Analyses

The outcome measure studied was 10-day all-cause mortality in relation to EDLOS. Mortality included both in-hospital deaths and deaths after discharge from the hospital. Patients who died in the ED were excluded. The total EDLOS was analyzed as a continuous variable, measuring the risk per hour. The potential confounding factors adjusted in the analyses were age, sex, comorbidities, shift, ESI score, and transport mode to the ED (EMS or other), indicating the severity of illness. The ESI scores were reduced to 3 variables (ESI 1-2, ESI 3, and ESI 4-5) due to the small number of ESI 1 and ESI 5 visits. To assess patient comorbidities, the updated Charlson Comorbidity Index (CCI) was calculated based on the ICD-10 coding algorithm developed by Quan et al.^21^^,^^24^

Continuous variables are presented as the mean with SD or the median with IQR. Nominal and categorical data are presented as numbers and percentages. The results of logistic regression analyses are presented as odds ratios (ORs) with 95% CIs. The statistical significance was tested using the chi-squared test, and *P* values < .05 were considered statistically significant.

Statistical analyses were performed using IBM SPSS Statistics (version 28.0.1.1).

## 3 Results

There was a total of 58,440 visits from 34,537 individual patients meeting the inclusion criteria during the study period. The 10-day mortality rate was 1.6% (n = 938), and the median time to death was 5 (IQR, 3-8) days. Of all the visits included, 33% (n = 19,242) were categorized as crowded. The median EDLOS was 5.0 (IQR, 3.7-6.6) hours, and there was no difference in the go-through times between conservative (n = 34,792) and operative (n = 23,610) specialties. The characteristics of the study population are presented in Table 1. The data on ESI scores were missing in 68 cases, EMS transport information in 29 cases, and the comorbidity index in 2 cases. Finally, multivariable logistic regression analyses yielded a total of 58,386 visits.

**Table 1:** Characteristics of the study population.

	All N = 58,440 n (%)	Crowded^a^ n = 19,242 n (%)	Noncrowded n = 39,198 n (%)
Age (y), mean (SD)	67.0 (20.2)	68.3 (19.0)	66.4 (20.7)
Male sex	28,242 (48.3)	9078 (47.2)	19,164 (48.9)
Shift			
Day	28,709 (49.1)	12,827 (66.7)	15,882 (40.5)
Evening	21,692 (37.1)	5744 (29.9)	15,948 (40.7)
Night	8039 (13.8)	671 (3.5)	7368 (18.8)
EMS transport	37,255 (63.7)	11,368 (59.1)	25,887 (66.0)
Missing	29 (0.0)	18 (0.1)	11 (0.0)
Triage acuity			
ESI 1-2	4417 (7.6)	1312 (6.8)	3105 (7.9)
ESI 3	52,881 (90.5)	17,606 (91.5)	35,275 (90.0)
ESI 4-5	1074 (1.8)	297 (1.5)	777 (2.0)
Missing	68 (0.1)	27 (0.1)	41 (0.1)
CCI			
None (CCI = 0)	32,893 (56.3)	10,429 (54.2)	22,464 (57.3)
Mild (CCI = 1-2)	15,932 (27.3)	5487 (28.5)	10,445 (26.6)
Moderate (CCI = 3-4)	6028 (10.3)	2085 (10.8)	3943 (10.1)
Severe (CCI ≥ 5)	3585 (6.1)	1241 (6.4)	2344 (6.0)
Missing	2 (0.0)		2 (0.0)
Death in 10 d	938 (1.6)	332 (1.7)	605 (1.5)
EDLOS (h), median (IQR)	5.0 (3.7-6.6)	5.8 (4.4–7.4)	4.6 (3.4–6.1)
Conservative^b^	4.9 (3.7-6.5)	5.6 (4.3-7.2)	4.5 (3.4-6.0)
Operative^c^	5.1 (3.6-6.8)	6.1 (4.6-7.9)	4.7 (3.4-6.3)
Other^d^	4.7 (2.0-6.8)	6.1 (5.1-7.4)	3.7 (1.6-6.1)

CCI, Charlson Comorbidity Index; EDLOS, emergency department length of stay; EMS, emergency medical services; ESI, Emergency Severity Index.

a Crowded = patient exposed to emergency department occupancy ratio > 90% during the visit.

b Conservative = medicine, neurology, psychiatry, and oncology (n = 34,792).

c Operative = orthopedic, surgery, ear-nose-throat, and gynecology (n = 23,610).

d Other = ophthalmology and undetermined (n = 38).

A prolonged EDLOS was associated with mortality only in the univariable analysis (OR for a 1-hour increase in EDLOS, 1.03 [95% CI, 1.01-1.06]; *P* = .007), but after adjusting for confounding factors, the results became nonsignificant (OR, 1.01 [95% CI, 0.99-1.04]; *P* = .383) (Table 2). Examining the crowded and noncrowded subgroups did not change the findings (Table 3).

**Table 2:** Association of emergency department length of stay with 10-day mortality.

	Univariate			Multivariable^a^		
	OR	95% CI	*P* value	OR	95% CI	*P* value
EDLOS (h)	1.03	1.01-1.06	.007	1.01	0.99-1.04	.383
Age (y)	1.05	1.05-1.06	<.001	1.05	1.04-1.05	<.001
Male sex	1.26	1.10-1.43	<.001	1.53	1.34-1.75	<.001
EMS transport	4.44	3.63-5.43	<.001	3.08	2.51-3.79	<.001
Triage acuity						
ESI 4-5	Ref			Ref		
ESI 3	2.78	1.24-6.21	.013	1.81	0.80-4.06	.153
ESI 1-2	4.93	2.17-11.22	<.001	3.52	1.54-8.06	.003
CCI						
None	Ref			Ref		
Mild	1.89	1.61-2.22	<.001	1.33	1.13-1.57	<.001
Moderate	2.96	2.46-3.58	<.001	1.85	1.52-2.23	<.001
Severe	4.77	3.93-5.79	<.001	3.86	3.17-4.69	<.001
Shift						
Morning	Ref			Ref		
Evening	0.81	0.70-0.94	.004	0.81	0.70-0.94	.005
Night	1.02	0.84-1.23	.840	0.98	0.81-1.19	.831

CCI, Charlson Comorbidity Index; EDLOS, emergency department length of stay; EMS, Emergency medical services; ESI, Emergency Severity Index; OR, odds ratio; Ref, reference.

a Analyses adjusted for age, sex, triage acuity, transport mode, shift, and CCI.

**Table 3:** Association of emergency department length of stay with 10-day mortality under different crowding conditions.

	Noncrowded (n = 39,198)			Crowded^b^ (n = 19,242)		
	OR	95% CI	*P* value	OR	95% CI	*P* value
EDLOS (h)						
Univariate	1.02	0.99-1.06	.199	1.04	1.00-1.08	.030
Multivariable^a^	0.99	0.96-1.03	.733	1.03	0.99-1.07	.111

EDLOS, emergency department length of stay; OR, odds ratio.

a Analyses adjusted for age, sex, triage acuity, transport mode, shift, and the Charlson Comorbidity Index.

b Crowded = patient exposed to emergency department occupancy ratio >90% during the visit.

## 4 Limitations

This was a single-center cohort study in a Finnish university hospital, which may decrease the generalizability of the results to other populations in other countries. It also limited the study sample size, combined with the current pandemic, which made it impossible to lengthen the study period.

As with any retrospective study, potential errors include incomplete or miscoded data. The number of missing values, however, was small, and only 54 visits (0.09%) were excluded from the final analyses because of it. One limitation of this study was that we were unable to separately analyze the waiting times, treatment times, or boarding times of the ED patients.

The authors recognize that some unadjusted patient-specific factors, such as the nature of the acute illness, will affect the length of ED stay and that more complicated cases may have longer go-through times. Street et al^25^ have shown in their study that among older patients, a need for a computed tomography scan or ultrasound or visiting during crowded hours increases the risk for prolongation of the ED stay. In addition, Dinh et al^26^ found that a CCI score of ≥5 affects the EDLOS even more than age. Most of these factors, such as patient acuity, age, and comorbidities, as well as ED occupancy, have been considered and handled as confounding factors in this study. Yet, there might still be other unknown factors, such as staffing resources or seasonal variability, which we did not adjust for.

Although mortality is probably the most important and frequently used outcome in studies regarding ED crowding and length of stay, it is not the only adverse event to take into consideration. In other studies, important findings regarding a longer EDLOS leading to in-hospital escalation of care and the need for rapid response teams,^27^ as well as in-hospital length of stay and costs,^28^ have been reported.

## 5 Discussion

According to this study, a longer EDLOS was not independently associated with the short-term mortality of patients admitted to the hospital. The observed increase in mortality was no longer significant after the adjustment for confounding factors. Thus, a longer EDLOS is likely to indicate the severity of illness and patient morbidity rather than being a risk factor for mortality, per se.

Due to the comprehensive records of deaths, we were able to obtain accurate data regarding the 10-day mortality rate, as it was not limited to in-hospital mortality. Because EDLOS and ED crowding are closely dependent on one another, we split the study population into 2 to evaluate how crowding affects the results, and there was no difference between the crowded and noncrowded subgroups.

Although this was the first study on the subject conducted in Finland, there are studies from other European countries with similar results. Wessman et al^29^ found that a prolonged EDLOS was not associated with 7-day mortality among admitted patients in a Swedish hospital. In Norwegian and French studies, there were no associations between EDLOS and mortality, but the studied outcome was 30-day mortality.^11^^,^^12^

There are some studies that have reached opposite results, demonstrating that longer ED stays affect patient outcomes. Jones et al^10^ studied over 5 million ED visits and found a linear correlation between prolonged waiting times and 30-day mortality. In other studies, if ED crowding was not adjusted, it is difficult to make robust conclusions about whether a longer stay merely represents crowded conditions in the ED or if it should be seen as an independent risk factor for patient mortality.^13^^,^^30^ Additionally, many of the studies showing an association between waiting times and mortality have focused on a specific patient group, such as the elderly or intensive care unit patients. For example, it has been shown that overnight stays in the ED increased in-hospital mortality and morbidity among older patients^31^ and that among critical trauma activation patients, a prolongation of the ED stay increased in-hospital mortality for each additional hour.^32^ In 2023, Lauque et al^33^ published a review study and meta-analysis, including 52 studies, and found an association between EDLOS and mortality only for stays over 24 hours regarding those patients admitted to the intensive care unit directly from the ED.

A review by Burgess et al^14^ analyzed 34 articles considering EDLOS and patient outcomes. The studies were heterogeneous in their settings and participants, and there was no clear conclusion to be made; half of the studies found an association, whereas the other half did not. This shows the complexity of the subject and the variety of confounding factors that can affect the results. For example, if the study population includes triage category 1 (or red) patients, who are the most critically ill, it may seem as if a shorter stay in the ED is associated with higher mortality.^29^^,^^34^ In our setting, we excluded resuscitation room patients, and we considered that they should be studied as their own entity.

Instead of the total EDLOS, some studies have measured the boarding time, ie, the time a patient stays in the ED waiting for an inpatient bed after the decision to admit has been made.^13^^,^^15^^,^^34^ A systematic review found no clear evidence that a longer boarding time increases in-hospital mortality, though a potential trend in this direction was observed.^16^ The boarding time is not the topic of this study, but it forms part of the total EDLOS and is most likely an important reason for the prolongation of patients’ stay in the ED.

Instead of mortality, longer stays in the ED can lead to other notable adverse events that must not be forgotten in the discussion. A significant part of the patients seen in the ED are the elderly. Several studies considering elderly ED patients have recognized risks related to a prolonged EDLOS, mortality, delirium, and prolonged hospitalization.^25^^,^^31^^,^^35^ The risk for hospital-acquired infections rises the longer the patient stays in the ED and in the hospital.^36^ In addition, inferior results have been reported in the treatment and recovery of patients presenting with a myocardial infarction,^37^ stroke,^38^ or intracranial hemorrhage^39^ if they were exposed to prolonged ED stays.

Even though this Finnish study found no clear association between EDLOS and mortality for an individual patient, the authors would like to highlight that prolonged stays lead to ED overcrowding and may thus increase mortality at the population level, as has been repeatedly demonstrated in prior studies.^2^, ^3^, ^4^, ^5^^,^^17^, ^18^, ^19^ It is reassuring for ED nurses and physicians that despite the long wait times, the quality of care mostly seems to meet patients' needs, at least from the perspective of short-term survival. However, the situation is neither desirable nor acceptable, considering the stress and discomfort it causes for both the employees and patients.

## Author Contributions

The study was designed by AE, JT, and JY-M. AE, JT, and JY-M obtained and processed the data. The statistical analyses were conducted by AE, JT, and HH, and all authors contributed to the data interpretation. AE, JY-M, and JT drafted the manuscript, and all authors were involved in the revision of the manuscript. The study was supervised by AP and TK. All authors have read and approved the final manuscript.

## Funding and Support

The study was funded by the Finnish Ministry of Health and Social Welfare via the Medical Research Fund of Kanta-Häme Central Hospital; the Competitive State Research Financing of the Expert Responsibility Area of Tampere University Hospital; Hauho Oma Savings Bank Foundation; and Renko Oma Savings Bank Foundation. None of them had a role in data collection, the analysis of the results, or the preparation of the manuscript.

## Conflict of Interest

All authors have affirmed they have no conflicts of interest to declare.
